# Absence of Middle East Respiratory Syndrome Coronavirus in Camelids, Kazakhstan, 2015

**DOI:** 10.3201/eid2203.151284

**Published:** 2016-03

**Authors:** Eve Miguel, Ranawaka A.P.M. Perera, Almagul Baubekova, Véronique Chevalier, Bernard Faye, Nurlan Akhmetsadykov, Chun Yin Ng, François Roger, Malik Peiris

**Affiliations:** Centre de Coopération Internationale en Recherche Agronomique pour le Développement, CIRAD, AGIR, Montpellier, France (E. Miguel, V. Chevalier, B. Faye, F. Roger);; The University of Hong Kong, Hong Kong, China (R.A.P.M. Perera, C.Y. Ng, M. Peiris);; LLP Antigen Research and Production Enterprise Antigen, Almaty, Kazakhstan (A. Baubekova, N. Akhmetsadykov);; Food and Agriculture Organization of the United Nations Camel Project, AlKharj, Saudi Arabia (B. Faye)

**Keywords:** Bactrian camels, dromedary camels, camels, camelids, central Asia, commercial flows, mountain chain, bats, Middle East respiratory syndrome coronavirus, MERS-CoV, viruses, Kazakhstan, respiratory infections

**To the Editor:** Middle East respiratory syndrome coronavirus (MERS-CoV) acquired from animals causes severe pneumonia in humans, with some chains of human-to-human transmission, leading to large outbreaks. MERS-CoV is a cause of concern for global public health. The only natural host of MERS-CoV identified so far is the dromedary camel (*Camel dromedarius*) (*1*,*2*), and transmission from camels to humans has been documented (*3*). The geographic distribution of MERS-CoV in dromedaries extends beyond the Arabian Peninsula (where human cases have been reported) to North and East Africa (where human cases have not been reported) (*2*,*4*). However, MERS-CoV from a camel in Egypt and MERS-CoV from a human were phenotypically similar in tropism and replication competence in ex vivo cultures of the human respiratory tract (*5*).

Our previous study demonstrated no evidence of MERS-CoV infection in Bactrian camels in Mongolia (*6*). The question whether MERS-CoV is endemic in camelids in Central Asia remains unanswered. MERS-CoV RNA was detected in swab samples from camels in Iran, which had been imported from Pakistan; however, where the infection was acquired is unclear (*7*).

In Asia, Kazakhstan is of particular interest because large populations of 2 major camelid species overlap: 90% Bactrian (Kazakh breed including 3 ecotypes) and ≈10% dromedary (Arvana breed from Turkmenistan) and their hybrids (*8*). To determine whether MERS-CoV is present in camelids in Kazakhstan, we conducted a seroepidemiologic survey. 

During February–March 2015, blood was collected from 550 female camels (455 dromedary, 95 Bactrian) (Figure) in 2 regions, Almaty and Shymkent, which differ in camelid density (0.034 and 0.20 camels/km^2^, respectively; http://www.stat.gov.kz). Dromedaries were sampled in the cities/villages of Kyzylorda (105 animals from 2 herds), Zanakorgan (35 animals from 1 herd), Sholakkorgan (110 animals from 2 herds), and Akshiy (205 animals from 4 herds). Bactrian camels were sampled in Sholakkorgan (40 animals from 1 herd) and Kanshengel (55 animals from 1 herd) (Figure). For dromedary camels, mean age was 6.1 years (SD 3–7 years) and mean herd size was 53.6 animals (SD 31–70); for Bactrian camels, mean age was 6.5 years (SD 5–8 years) and mean herd size was 48.6 animals (SD 40–55). Serum samples were tested for MERS-CoV antibodies at a screening dilution of 1:20 by using a validated MERS-CoV (strain EMC) spike pseudoparticle neutralization test (*9*). Positive and negative controls were included in each run. Absence of positivity for any sample indicated a lack of recent or past MERS-CoV infection.

**Figure:**
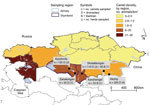
Density of camelids in Kazakhstan (extracted from the Ministry of National Economy of the Republic of Kazakhstan Committee on Statistics, Department of Statistics; http://www.stat.gov.kz) and specimen collection for detection of Middle East respiratory syndrome virus, by species and region, 2015.

Two randomly selected samples each from dromedaries from Kyzlorda, Zanakorgan, and Akshiy and Bactrians from Sholakkorgan and Kanshenegel were tested for neutralizing antibody to bovine coronavirus (*9*). All 10 samples were seropositive, as has been reported for Bactrian camels in Mongolia and the Middle East (*6**,**9*).

Given the uniformly high seroprevalence of MERS-CoV infection among dromedaries in Africa and the Arabian Peninsula, the lack of infection in dromedaries in southern Kazakhstan was surprising. Because genetically diverse MERS-CoV from Africa remains antigenically conserved with viruses from the Arabian Peninsula, the lack of antibodies is probably not explained by antigenically divergent strains (*9*). Feral dromedaries in Australia, which originated from animals imported from Afghanistan or Pakistan during 1840–1907, are also seronegative for MERS-CoV (*10*). In contrast, bovine-like coronavirus seems to be present in dromedaries everywhere (including Kazakhstan and Australia). 

Our study was limited by sample size and by geographic coverage. Of the ≈180,000 camels in Kazakhstan, we studied camelids from only 2 of the 13 provinces. No samples were collected from the western part of the country near Turkmenistan, where dromedaries are also common.

Dromedaries are clearly a natural host of MERS-CoV. However, the finding that MERS-CoV is not endemic in dromedaries in all geographic regions suggests the possibility that dromedaries may not be the ultimate natural reservoir (i.e., the long-term host of a pathogen of an infectious disease). Topography (i.e., mountain chains) may limit camel movements from the Middle East or Africa to Central Asia, although such interchange certainly occurred centuries ago as a consequence of the silk-trade routes through southern Kazakhstan. The only known recent imports to Kazakhstan are dromedaries (Arvana breed), brought from Turkmenistan for cross-breeding with Bactrians to improve milk production (*8*). The findings that MERS-CoV is not universally endemic in dromedaries raises the hypothesis that certain species of bats or some other animal, the environment, or both, may constitute a maintenance community and be the true natural reservoir of MERS-CoV and that the virus spills over to camels and is maintained within camels for varying periods of time. Further studies on the epidemiology of MERS-CoV infection among camelids from central Asia are warranted.
